# Differences in Olfactory Discrimination, but Not Sensitivity, Between African Savanna and Asian Elephants

**DOI:** 10.1002/ece3.71896

**Published:** 2025-08-11

**Authors:** Melissa H. Schmitt, Matthew S. Rudolph, Sarah L. Jacobson, Joshua M. Plotnik

**Affiliations:** ^1^ Department of Biology University of North Dakota Grand Forks North Dakota USA; ^2^ School of Biology and Environmental Sciences University of Mpumalanga Nelspruit South Africa; ^3^ Department of Psychology, Hunter College City University of New York New York New York USA; ^4^ Department of Psychology, The Graduate Center City University of New York New York New York USA

**Keywords:** cognition, evolutionary ecology, foraging ecology, olfaction, sensory abilities

## Abstract

While African savanna and Asian elephants split between 4.2 and 9 MYA, they are often regarded as one united group, ‘elephants.’ This is surprising because, while both are keystone species in their respective habitats, each faces different environmental pressures and has rarely been compared experimentally. In general, African savanna elephants must locate resources that vary spatially and temporally across patchy savannas, while Asian elephants do so within dense, high‐biodiversity forests. Both species use olfaction to guide decision‐making; however, considering their ecologies, we hypothesize that their olfactory abilities differ. Thus, we investigated the sensitivity limits and discrimination abilities of both savanna and Asian elephants' olfactory systems, and changes in these limits in a complex odor environment. We employed two odor‐based choice experiments, using *c* *is‐3‐Hexenyl acetate*—a common green leaf volatile that is emitted by plants globally—as a target odor. While both species correctly detected a target odor, albeit at different concentrations—savanna elephants detected it at 50 parts per million (ppm) and Asian elephants at 100 ppm—only the savanna elephants' limit changed (to 1000 ppm) in the complex odor environment. While we were limited by a small sample size (i.e., *n* = 5 for each species), our data suggest that there may be differences in the olfactory abilities of these two elephant species.

## Graphical abstract

While African savanna and Asian elephants split between 4.2 and 9 MYA, they are often regarded as one united group, ‘elephants,’ even in the scientific literature. This is surprising, because while both are keystone species in their respective habitats, each faces different environmental pressures and has rarely been compared experimentally. Thus, we investigated the sensitivity and detection limits of both savanna and Asian elephants' olfactory systems, and changes in these limits in a complex odor environment using two odor‐based choice experiments. While both species correctly detected a target odor—savanna elephants detected it at 50 parts per million (ppm) and Asian elephants at 100 ppm—only the savanna elephants' limit changed (to 1000 ppm) in the complex odor environment. While we were limited by a small sample size, our data suggest that there may be differences in how these two species of elephants detect odors.

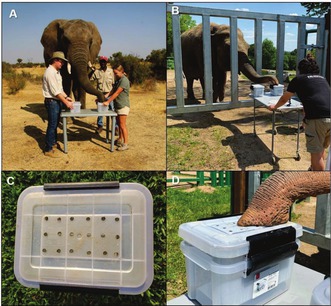

## 1 Introduction

Phenotypic comparisons among closely related and relatively recently diverged species (e.g., chimpanzees ( *Pan troglodytes* ) and bonobos ( *Pan paniscus* ); Prüfer et al. 2012) are often made to understand the role that environmental pressures play in shaping differences in the evolution of behavior (Boesch et al. 2002; de Waal 2013). While these comparisons are prolific in the literature through observational studies (e.g., Brown et al. 2004; de Silva and Wittemyer 2012; Stoeger and de Silva 2013), experimental assessment of the behavioral differences between Asian and African elephants is lacking. This is surprising because they are morphologically similar but have a more distant phylogenetic split than chimpanzees and bonobos, species that are frequently used in comparative behavioral research. African and Asian elephants genetically diverged 4.2–9 MYA (Rohland et al. 2010). Since then, African populations have split into the extant savanna elephants ( *Loxodonta africana* ) and forest elephants ( *Loxodonta cyclotis* ), while Eurasian populations diverged into the extant Asian elephant ( *Elephas maximus* ) and extinct mammoth (*Mammuthus primigenius*) (Rohland et al. 2010). Asian and African savanna elephants (hereafter: savanna elephants) are sometimes lumped together or considered analogous, which has led to conservation actions designed for one species being applied to the other despite potential differences between the species (e.g., the use of bee‐hive fences to deter Asian elephants from crop raiding, which was originally designed for African savanna elephants (Dror et al. 2020)). The analogous perception of the two species likely stems from their broadly similar morphology, comparable foraging habits, and their roles as ecosystem engineers and keystone species in their respective habitats (de Silva et al. 2017; de Silva and Wittemyer 2012). However, what is often overlooked is that these species face markedly different physical, environmental, and anthropogenic challenges, which may play a key role in shaping differences in their behavior (Loarie et al. 2009a, 2009b; Sukumar 2006).

Although both species fill comparable niches as the largest generalist herbivore in their respective ecosystems (de Silva and Wittemyer 2012), there are some key ecological differences between their environments. Savanna elephants live in a range of habitats but are primarily found in mesic to arid woodlands (i.e., land covered with woody vegetation) and savannas (i.e., areas characterized by a mosaic of trees and grasses) (Grubb et al. 2000). While Asian elephants also inhabit a variety of habitats, including forests, shrubland, and grassland, they are considered to be forest edge specialists with a preference for a combination of natural forest and secondary vegetation (de la Torre et al. 2021; Evans et al. 2020; Fernando et al. 2008). On average, the habitats that savanna elephants live in have lower quality food (Net Primary Production (NPP) < 700 g C m^−2^) compared to the habitats where Asian elephants occur (< 1200 g C m^−2^) (Cramer et al. 1999). Another key difference among their habitats is the level of floristic diversity, which provides vastly different numbers of potential food options. The ecoregions where savanna elephants occur have ~500–3000 species of vascular plants, while the ecoregions where Asian elephants occur include between 1000 and 5000 species (Kier et al. 2005). Thus, Asian elephants need to find key food resources in floristically more complex environments compared to savanna elephants. Moreover, food resources are not evenly distributed across the landscape and are also subject to dramatic changes in availability as a result of seasonality (Anderson et al. 2016; Scholes et al. 2003). This is particularly important for savanna elephants that often live in ecosystems which are characterized by pronounced wet and dry seasons (Scholes et al. 2003; Young et al. 2009). During the dry season in particular, high‐quality food resources are less available than they are in the wet season and are often spread across the landscape, mixed in with lower‐quality resources (Davies et al. 2016; Skidmore et al. 2010).

With respect to foraging behavior, although both species are considered to be generalist mixed feeders (i.e., they consume both woody plants and grasses), they do exhibit a degree of selectivity (Koirala et al. 2016; Owen‐Smith and Chafota 2012; Schmitt et al. 2020; Shrader et al. 2012). The proportional contribution of woody plants vs. grasses to both species' diets can vary both temporally (e.g., across seasons) as well as spatially depending on vegetation availability within their local habitats (Codron et al. 2006, 2012; Koirala et al. 2016; Sukumar 1990, 2006). Being megaherbivores (species > 1000 kg in mass), their large body size results in both species having high absolute intake rate requirements (e.g., > 150 kg of vegetation per day: Vancuylenberg 1977; Wyatt and Eltringham 1974). Given this, locating resources that vary in quality and quantity across the landscape has important implications for shaping elephant behavior. For example, savanna elephants have much larger maximum home range sizes (e.g., 3700 km^2^) than Asian elephants (e.g., 1000 km^2^) (Delsink et al. 2013; Sukumar 2006). This difference is largely a result of differences in resource availability (e.g., plant productivity) and human landscape modifications that encroach into and fragment existing habitat (Loarie et al. 2009a, 2009b; Sukumar 2006). Ultimately, the two elephant species must locate resources in habitats that differ in resource availability, quality, and spatial distribution.

To guide their behavioral decision‐making within these heterogeneous environments, both elephant species can use cues gathered from their environment. Numerous studies have reported the importance of olfactory cues for social communication and food selection in Asian and savanna elephants, and that their sense of smell may play an important role in physical and social decision‐making (Arvidsson et al. 2012; Hollister‐Smith et al. 2008; Plotnik et al. 2014; Rasmussen and Wittemyer 2002). While olfactory behavioral choice experiments have been performed with both savanna and Asian elephants (Bester et al. 2023a, 2023b; Finnerty et al. 2024; McArthur et al. 2019; Nevo et al. 2020; Plotnik et al. 2019; Schmitt et al. 2018; Valenta et al. 2021; Wood et al. 2022), there have been no studies comparing the olfactory abilities of the two species. Investigating differences in their olfactory abilities, considering how important olfaction is to both species, may provide insight into whether and how environmental differences in which these species evolved may have shaped variation in the relationship between the elephants' olfactory capacities and their foraging behavior. Thus, for the first time, we investigated whether there are differences in how Asian and savanna elephants detect odors. We were specifically interested in understanding the limit of both species' capacity for olfactory detection, as well as whether those limits fluctuated in a complex odor environment. To do this, we used two odor‐based experiments. The first experiment aimed to determine a limit for olfactory sensitivity under controlled conditions. Olfactory sensitivity is measured by determining the lowest concentration of an odorant that a subject can detect (Van Gemert 2003). The second experiment aimed to determine whether this threshold varied when a masking odor was present, to mimic conditions of wild foraging in a complex odor environment (i.e., floristically diverse environments). Experiment 2 therefore tested olfactory discrimination, requiring the elephants to discriminate between the target odor and the potential masking odor. Olfactory discrimination is the ability to respond differentially to the successive presentation of nonidentical odorants (Hedner et al. 2010).

For the ‘sensitivity threshold’ experiment (Experiment 1), we hypothesized that savanna elephants would perform better than Asian elephants because savanna elephants must locate resources that are more patchily distributed across their habitats than those in which Asian elephants live (Davies et al. 2016; Skidmore et al. 2010). However, it is possible that both species have similar overall olfactory abilities, which is likely rooted in their shared evolutionary history. For the ‘detection in complex environments’ experiment (Experiment 2), we hypothesized that Asian elephants may out‐perform savanna elephants because they must locate target food resources that are mixed into a high diversity of other plants in structurally dense habitats. This would result in more complex odor environments, compared to those in which savanna elephants live. Alternatively, the ability to detect target food items in complex odor environments may be similar for both species due to their shared ancestry. Any differences in olfactory discrimination between the two species are likely the result of the different ecological and environmental factors each species faces in their respective habitat, and thus relatively recent evolutionary pressures that have resulted in divergent olfactory abilities.

## 2 Methods

Using similar procedures conducted in previous research on elephant olfaction (e.g., Bester et al. 2023a, 2023b; McArthur et al. 2019; Plotnik et al. 2013, 2014, 2019; Schmitt et al. 2018; Wood et al. 2022), we aimed to compare the olfactory capabilities of savanna and Asian elephants with respect to their abilities to detect differing emissions of *cis‐3‐Hexenyl acetate* (Sigma‐Aldrich, natural ≥ 95% FG, CAS 3681‐71‐8). *cis‐3‐Hexenyl acetate* is a common green leaf volatile that is emitted by plants globally, including by plants consumed by both species of elephants (Crespo et al. 2013; Koirala et al. 2016; McArthur et al. 2019; Schwarz et al. 2020; Soler et al. 2011). Thus, we were confident that both elephant species could detect this Volatile Organic Compound (VOC). To compare the olfactory capabilities of savanna and Asian elephants, we conducted two odor‐based object choice experiments. In the first experiment, we aimed to assess the sensitivity threshold of Asian and savanna elephants using serial dilutions of *cis‐3‐Hexenyl acetate* ranging from 100,000 ppm to 5 ppm (Table 1). In the second experiment, we aimed to understand how this threshold varied when a masking odor was present to mimic conditions of foraging in a complex odor environment. The range of concentrations of *cis‐3‐Hexenyl acetate* was based on previous research that has shown Asian elephants can successfully detect and discriminate a target odor from 100,000 ppm down to 10,000 ppm (Arvidsson et al. 2012; Rizvanovic et al. 2013). Both of our experiments were run in the same manner; however, in the second experiment, an additional odor was added to each bucket in a separate vial to act as a masking odor to increase the complexity (noise) of the background odor (see below).

**TABLE 1:** Serial dilutions of *cis‐3‐Hexenyl acetate* used in both experiments represented by ppm of *cis‐3‐Hexenyl acetate* suspended in liquid paraffin.

Treatment	ppm
A	100,000
B	50,000
C	10,000
D	5000
E	1000
F	500
G	100
H	50
I	10
J	5

### 2.1 Subjects

**FIGURE 1:**
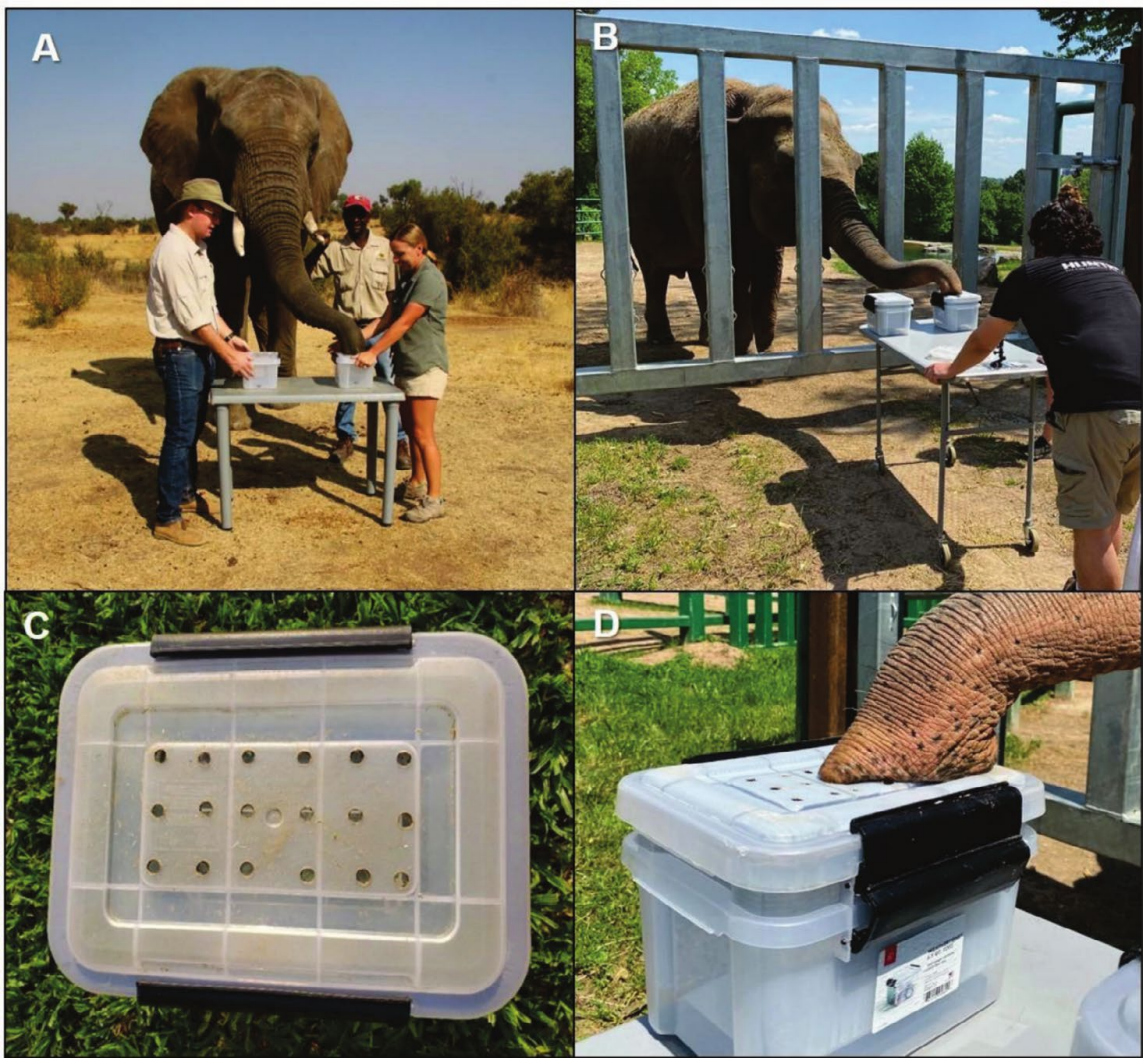
Panel (A) depicts the testing setup conducted with free‐contact savanna elephants, while (B) shows the rolling table testing setup conducted with Asian elephants in protected contact. Free contact allows for direct interaction with the elephants; protected contact means a barrier exists between humans and the elephants. *N.B*. experimental testing occurred in an open‐air barn with the Asian elephants. This image was taken just outside the barn for demonstration purposes only. Panel (C) shows an example of the testing bucket in which a vial of either the target odor or control odor is hidden, and (D) illustrates an Asian elephant smelling through the holes in the lid during the experimental procedure.

The experiments were conducted with savanna elephants in September 2019 at the Adventures with Elephants facility near Bela Bela, Limpopo Province, South Africa. For all trials, professional elephant handlers were present to ensure the comfort and safety of the elephants. We used five semi‐tame, wild foraging, adult individuals between 20 and 25 years old (three females, two males). Neither male elephant was in musth during the duration of our study. The experiments with Asian elephants were conducted between October 2020 and July 2021 at the Rosamond Gifford Zoo in Syracuse, NY, USA. We studied five resident Asian elephants between 24 and 53 years old (five females). Both experiments for the savanna elephants were conducted in a free‐contact environment (as pictured in Figure 1), with two people holding buckets on a stationary table and a third person—a handler—standing beside the elephant to give commands. However, a protected‐contact testing procedure was used when testing the Asian elephants, following the zoo's elephant husbandry and handling guidelines (Figure 1). This protected‐contact procedure adapted the methods of Plotnik et al. (2019), which used a sliding table to present the elephants with the buckets; however, there were always two handlers present to oversee the experimental procedures and give commands to the elephants. To ensure that the handlers did not cue the elephants to the target odor, the handlers were naïve to the concentrations in each bucket. While we acknowledge that the odorants linked to handlers and each testing setting may vary, we took multiple steps to minimize the possibility of creating varying ambient odor environments. While we could not control the entire odor profile of the handlers and there may be international differences in the soaps or washing powders they used, we instructed the handlers to carry out their daily activities as usual (i.e., without changing fragrant items such as creams or soaps). As an additional precaution to limit competing odors, we conducted our experiments in high airflow areas (i.e., outdoors for savanna elephants or in an open‐air barn with the doors open for Asian elephants). Moreover, given that the animal care teams had been working at their respective facilities for years, the elephants were likely habituated to the odors of their respective teams.

### 2.2 Experimental Design

The Asian and savanna elephants in our study were tested using the same basic experimental setup for both experiments. Similar to prior studies (e.g., McArthur et al. 2019; Plotnik et al. 2013, 2014, 2019; Schmitt et al. 2018; Wood et al. 2022), we presented the elephants with a binary choice using buckets in which either the target odor or control odor was hidden (as well as the addition of the masking odor in the case of Experiment 2). The buckets were clear 6.15 L plastic totes, measuring 29.2 cm × 21.5 cm × 16.5 cm with latching lids. The lids had 18 holes drilled into the top to allow airflow. Holes were drilled in a rectangular grid fashion across a 120 cm^2^ portion of the lid. In the savanna elephant tests, handlers held the buckets on a stationary table, while in the Asian elephant tests, the buckets were secured to the table by nesting them inside a second bucket that was bolted to the rolling table (Figure 1). The bucket bolted to the table acted simply as a brace, and there was no hindrance to airflow for the buckets nested within them in which the odorants were placed. To avoid any selection bias based on the handlers holding the buckets in place for the savanna elephant experiments, handlers were rotated randomly. Additionally, the location of the target odor (i.e., in the left or right bucket) was randomized using a random number generator to ensure that side bias did not influence the animals' choices. To ensure that each elephant did not observe the experimental setup before each trial began, a professional handler instructed the elephants to face away (180°) from the testing arena before buckets were presented; in this position, it was impossible for the elephants to see the placement of the bins. Once the vials holding the target and control odors (and masking odor in the case of Experiment 2) were placed inside each bin, the bins were arranged side‐by‐side on a table. The elephant was then instructed to turn, face forward, and to “smell” the bins. At this point, the elephant stepped up to the bins and placed their trunk on each holey lid and inhaled the odors from each bucket. As per a number of previous studies (Bester et al. 2023a; McArthur et al. 2019; Plotnik et al. 2013, 2014, 2019; Schmitt et al. 2018), each elephant was able to smell or touch each bucket when presented, but could not open or reach inside them. The elephants had 10 s to touch and smell both buckets on the table. If the elephants smelled both buckets in 10 s, the elephants and the buckets were separated at the 10 s mark and then buckets were re‐presented after 3 s so that the elephants could make a choice. In the case of the savanna elephants, we instructed them to remove their trunks from the buckets at the 10 s mark; however, due to the protected‐contact requirements associated with the Asian elephants' environment, we rolled the table with the buckets away from the elephants instead of instructing them to remove their trunks. Each elephant indicated which bucket contained the target odor by touching or tapping on it. We considered an elephant to have made their choice by tapping or touching any part of the first bucket they interacted with after the buckets were re‐presented. After the elephant made their choice, they were rewarded with a food reward if correct. The lids and buckets were cleaned with a wet cloth with isopropyl alcohol in between each trial to ensure that the elephants could not use mucus deposited during previous trials (Bester et al. 2023a, 2023b). While we did not use a separate cloth for each trial, the buckets were wiped down multiple times to reduce any potential identifying odor cues. The buckets were thoroughly cleaned with soap and water between elephants to limit any potential bias.

For our second experiment, we investigated the use of olfaction during foraging in a complex odor environment, and used *1‐Nonanol* (Sigma‐Aldrich, purum ≥ 98%, CAS 143‐08‐8), as a masking odor. Nonanol is commonly found in the environment (Helms et al. 2017; Wonglom et al. 2020), but is not something that has a known positive or negative association for either species of elephant. The concentration of this odor remained constant throughout the experiment. Its concentration was set to 100,000 ppm, which is relatively high (Peixoto et al. 2018), to determine how well each species can detect weaker odors when there are other, potentially highly concentrated, odorants in the environment.

### 2.3 Training

Prior to running the experiments, both Asian and savanna elephants were trained in the same fashion to select a high concentration of *cis‐3‐Hexenyl acetate* hidden inside a bucket. To teach the elephants the nuances of a behavioral choice experiment, they were initially trained to detect a food item in one of the buckets. When the elephants chose the bucket containing the food item ≥ 8/10 times in two consecutive sessions of ten trials, they moved on to training to detect *cis‐3‐Hexenyl acetate*. Chemical detection training consisted of the same setup as testing, although the buckets were transparent, visually identical, and contained either a vial of 150,000 ppm *cis‐3‐Hexenyl acetate* or the same suspension fluid (i.e., liquid paraffin) with no target odor. Once the elephants successfully chose the bucket containing the target odor ≥ 8/10 times in three consecutive sessions of ten trials each, they moved on to experimental trials. For all training sessions, elephants were rewarded every time they correctly identified the target odor. Although the presentation of the odors differed between the two species due to protected vs. free contact conditions under which the species were kept (see above), all individuals reached criteria indicating that they understood the experimental setup. This rigorous training regime ensured that the elephants were motivated to participate and delivered consistent results in detecting olfactory information. The training also allowed the elephants to learn (a) the task procedure to select one of two buckets after first being presented with two options and then having to make a choice, and (b) to detect and identify a 150,000 ppm concentrated solution before we started testing them on more diluted solutions.

### 2.4 Testing

For both experiments, each elephant was tested for six trials of one target odor concentration (i.e., treatment) per session. The 10 different concentrations (see Table 1) were presented in a random order over the sessions of each experiment, which resulted in each elephant experiencing 60 trials per experiment (i.e., 6 trials × 10 concentrations). This resulted in each elephant receiving 20 total sessions of testing across both experiments (see Tables S1–S4 for individual elephant choices for each treatment for both experiments). For both groups of elephants, no more than two concentrations were tested per day (i.e., two sessions). We used two testing times per day: one at 9 am and the other at 12 pm. Each elephant had a minimum gap of 3 h between testing sessions. The two experiments were conducted with the savanna elephants over a 10‐day period and over the course of 30 days for the Asian elephants. For the savanna elephants, one individual (elephant #2) refused to participate in two sessions for Experiment 1 (i.e., 100,000 ppm and 5000 ppm) and two sessions for Experiment 2 (i.e., 100,000 ppm and 10,000 ppm); thus, these data are missing from our analyses (see Supporting Information Tables S1 and S3). To ensure hunger levels did not influence diet selection, the elephants were able to feed for 1 h prior to testing.

### 2.5 Statistical Models

For each experiment, the elephants were tested against 10 different concentrations of the target odor six times. The results from all trials from both experiments were analyzed using generalized estimating equations (GEEs). We treated individual elephants as the subjects for repeated measures in GEEs because of the potential non‐independence of our data, which could stem from an individual's growing experience over repeated trials. Furthermore, GEEs use a population‐level approach based on a quasi‐likelihood function, which delivers population‐averaged estimates of the parameters. In addition, the coefficients of GEE regressions are marginal effects (i.e., the effects average across all the subjects in the data: Wang 2014). Thus, in our case, GEEs modeled the number of times the elephants made the correct choice (i.e., selected the bucket with the target odor) compared to a 50% distribution expected under random selection for a given choice. Our model used an exchangeable correlation matrix and a binomial error distribution with a logit link function. We ran separate models for each species and each experiment (i.e., four separate models). To explore whether sensitivity varied across treatments, we considered the chemical concentration (treatment) as the independent variable and the successful detection of the target odor as the dependent variable.

## 3 Results

### 3.1 Experiment 1

**FIGURE 2:**
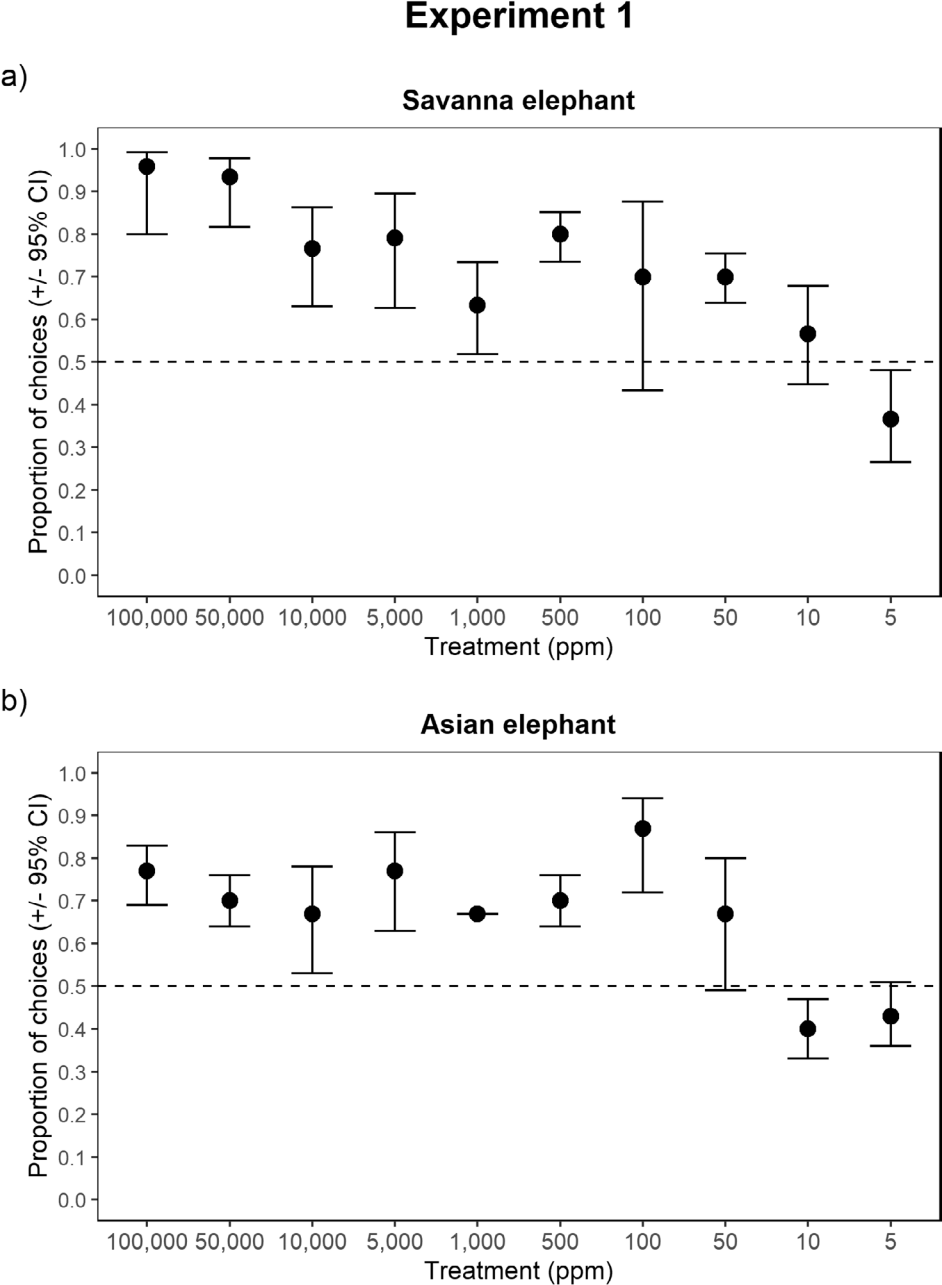
Proportion of choices in which the target odor was selected as a function of diminishing concentration (ppm) of the target odor. Panel (a) reflects savanna elephant detection abilities while panel (b) reflects Asian elephant detection abilities. Marginal means (±95% Confidence Intervals) of the proportion of selection of a given treatment are plotted. If there is no overlap of the 95% CI with the 0.5 expectation (i.e., random selection), this indicates significant selection for the target odor. If there is overlap with the 0.5 expectation (i.e., random selection), this indicates no significant selection for the target odor.

The concentration of *cis‐3‐Hexenyl acetate* presented to both savanna and Asian elephants significantly influenced their choice (savanna: GEE: *χ* ^2^ = 53.291, *p* < 0.0001, Asian: GEE: *χ* ^2^ = 101.558, *p* < 0.000). Across 7 of the 10 concentrations, both savanna and Asian elephants correctly detected *cis‐3‐Hexenyl acetate* (Figure 2a,b). Both savanna and Asian elephants were unable to detect the target odor in the treatments with the weakest concentrations of the target odor—10 ppm and 5 ppm, respectively. However, the two species differed with respect to the third treatment in which they were unable to discern the target odor. Savanna elephants were unable to significantly identify the target odor at 100 ppm, but were able to detect the target odor at 50 ppm, which is one treatment level more diluted than 100 ppm. By contrast, Asian elephants were able to identify the target odor at 100 ppm, but not at 50 ppm. While the significant detection for this third treatment differed between the two species, the threshold drop‐off points in sensitivity occurred in treatments that were within the same magnitude of concentration (i.e., 50 ppm is half the concentration of 100 ppm).

### 3.2 Experiment 2

**FIGURE 3:**
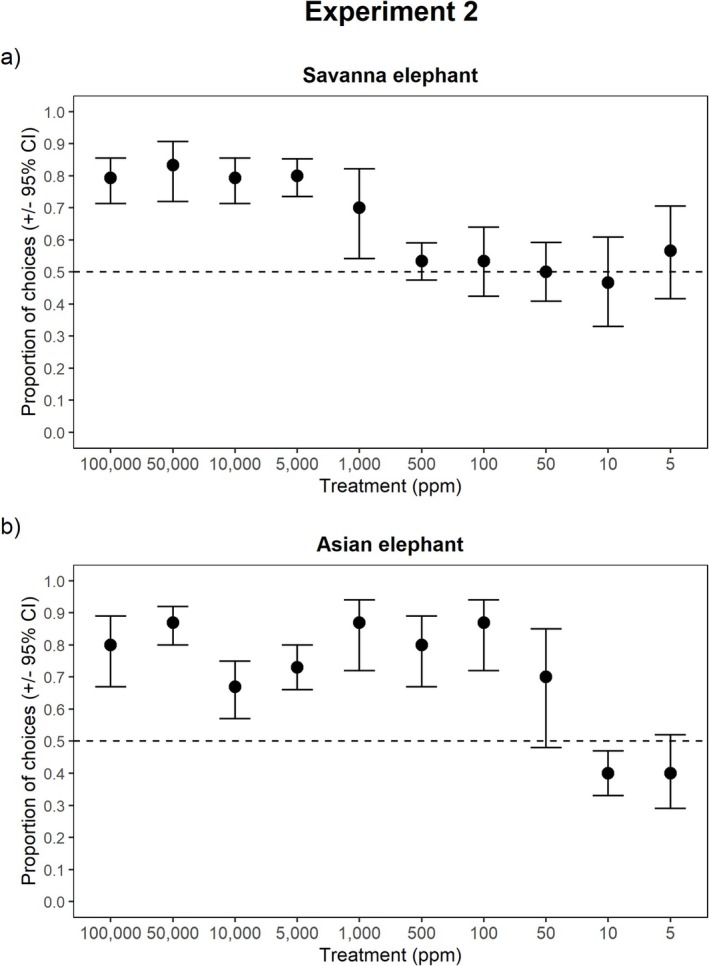
Proportion of choices in which the target odor was selected as a function of diminishing concentration (ppm) of the target odor in the presence of a masking odor. Panel (a) reflects savanna elephant detection abilities, while panel (b) reflects Asian elephant detection abilities. Marginal means (±95% Confidence Intervals) of the proportion of selection of a given treatment are plotted. If there is no overlap of the 95% CI with the 0.5 expectation (i.e., random selection), this indicates significant selection for the target odor. If there is overlap with the 0.5 expectation (i.e., random selection), this indicates no significant selection for the target odor.

The concentration of *cis‐3‐Hexenyl acetate* presented to both savanna and Asian elephants when in the presence of a masking odor significantly influenced detection (savanna: GEE: *χ* ^2^ = 52.303, *p* < 0.0001, Asian: GEE: *χ* ^2^ = 910.313, *p* < 0.0001). Savanna elephants were able to detect the target odor in 5/10 treatments, while Asian elephants maintained their ability to detect the target odor in 7/10 treatments (Figure 3a,b). In the presence of the masking odor, the sensitivity threshold for savanna elephants was 1000 ppm, while for Asian elephants it remained 100 ppm.

## 4 Discussion

While savanna and Asian elephants have historically been categorized as similar species both morphologically and behaviorally, our study is the first, to our knowledge, to make a direct comparison between their olfactory sensory abilities. We have shown that elephants have an olfactory sensitivity limit lower than previously determined (Arvidsson et al. 2012; Rizvanovic et al. 2013), ≥ 50 ppm for savanna elephants and ≥ 100 ppm for Asian elephants. When analyzed in a complex odor environment, these limits have disparate fluctuations for each species. In our study, the addition of a complex odor environment decreased savanna elephants' sensitivity and discrimination ability for odor detection by an order of magnitude (from ≥ 50 ppm up to ≥ 1000 ppm). However, the addition of a complex odor environment for Asian elephants did not impact their ability to detect the target odor. The similar initial sensitivity of 50 ppm and 100 ppm, which are within the same order of magnitude of concentration, could potentially be due to savanna and Asian elephants' shared ancestry. The differential results we observed in Experiment 2 could reflect more recent evolutionary adaptations that were driven by different ecological and environmental pressures, such as differences in plant diversity and nutritional quality.

While our results are limited by small sample sizes (i.e., 5 individuals per species) our results suggest that both savanna and Asian elephants have a sensitivity limit to their olfactory capabilities ≥ 100 ppm. This highlights the importance of olfaction as a key sense that both savanna and Asian elephants use to locate resources that vary both spatially and temporally. For all herbivores, the ability to detect a salient cue in their environment can be integral to their foraging success (Bedoya‐Pérez, Isler, et al. 2014; Bedoya‐Pérez, Issa, et al. 2014; Helle 1984). Each bite a herbivore takes represents a decision about what plant or plant part to eat (Senft et al. 1987). Thus, olfactory cues can be a key indicator that herbivores use to inform their decisions. For species such as savanna and Asian elephants that forage for a majority of a 24‐h cycle (Owen‐Smith 1988), live in ecosystems containing hundreds or thousands of different species of plants (Kier et al. 2005), and consume vast quantities of vegetation (Vancuylenberg 1977; Wyatt and Eltringham 1974), enhanced localization and detection can have a multiplicative benefit on the animal's time and energy budgets.

The greater the level of sensory information available to an animal, the faster they can decide whether to ingest a food item or not (Castellano and Cermelli 2015; Sulikowski 2017). Thus, enhanced detection down to 100 ppm of a target odor could provide elephants with additional olfactory evidence on which to base their foraging decisions. Plant species vary not only in their abundance but also in their nutritional quality, and both of these factors can vary across a seasonal cycle (Owen‐Smith 1994; Schmitt et al. 2020; Stears and Shrader 2020). Thus, for herbivorous mammals, olfaction can be a useful tool to locate and assess variable resources (Bester et al. 2023a; Finnerty et al. 2022, 2024; Kos et al. 2012; McArthur et al. 2019; McNaughton et al. 1988; Rode et al. 2006; Stutz et al. 2015, 2016). For example, reindeer ( *Rangifer tarandus* ) were able to distinguish good and poor lichen sources via olfactory cues below 90 cm of snow (Helle 1984), and savanna elephants can detect differences in sugar content of fruits from marula trees, *Sclerocarya birra* (Nevo et al. 2020). Moreover, Bester et al. (2023a) demonstrated that savanna elephants show varied responses (i.e., neutral vs. avoidant) to differing concentrations of volatile monoterpenes that are found in the plants in their environment, indicating the importance of olfactory sensitivity for foraging decision‐making. Beyond making decisions at a patch scale, herbivores also make foraging decisions at larger spatial scales (Ritchie and Olff 1999). For example, both swamp wallabies ( *Wallabia bicolor* ) and greater sage‐grouse ( *Centrocercus urophasianus* ) use olfactory cues emitted from target plant species to locate resources across the landscape (Finnerty et al. 2017; Frye et al. 2013; Stutz et al. 2015, 2016). Consequently, being able to detect 100 ppm of a target odor could help an herbivore identify important VOCs that are far away because the concentration of many VOCs declines with distance from the source (Fall 1999; Tigney 1991; Shuai et al. 2018). This detection ability may allow animals to make foraging or movement decisions at larger spatial scales, potentially optimizing energy budgets by reducing the need for costly exploratory movements. This would be particularly important for species occurring in seasonal environments, such as African savannas, where resource quality and quantity vary dramatically. Our findings suggest a crucial direction for future research—investigating how olfactory sensitivity shapes the decision‐making of large herbivores at the landscape scale.

Target plant species can also be hidden among an array of other plant structures or within an environment with a diversity of species, making desired items difficult to locate visually. However, herbivores can use olfactory cues emitted from target species to locate them in complex odor environments (McArthur et al. 2019; Stutz et al. 2015, 2016). Interestingly, when we tested whether the sensitivity threshold for both Asian and savanna elephants varied when a masking odor was present, we found disparate results between the two species. The presence of a masking odor did not impact the ability of Asian elephants to detect the target odor; however, its presence shifted the sensitivity threshold for savanna elephants by an order of magnitude, whereby they could only now detect the target odor when it was 1000 ppm instead of 100 ppm. Our results suggest that a complex odor environment does not affect the ability of Asian elephants to discriminate, while it does appear to influence the discrimination abilities of savanna elephants. This difference may reflect evolutionary adaptations to their respective natural histories and foraging environments, which could have shaped differences in sensory abilities. Dense forests with covered canopies, where Asian elephants are naturally found (Blake and Hedges 2004), have between 1000 and > 5,000 species of vascular plants (Kier et al. 2005). Several studies have demonstrated that forest canopies have reduced VOC diffusion (Denmead 1984; Denmead and Bradley 1985) and trap odors below the canopy (Raupach 1989). This incubator effect, coupled with high vegetation cover and high species diversity that characterize the habitats where Asian elephants occur (Letourneau 1987; Thomas et al. 1999; Wiesmair et al. 2017), could result in habitats with highly complex odor environments. Therefore, Asian elephants may need to be able to distinguish trace amounts of a target odor among many other odors while foraging, explaining their performance in the discrimination experiment. While both savanna and Asian elephants forage selectively (i.e., they do not eat everything that is available to them—Owen‐Smith and Chafota 2012; Sukumar 1990), it is possible that Asian elephants perform better with complex background odors compared to savanna elephants as a result of their high plant biodiversity and high odor complexity environment. Thus, each species' performance is potentially indicative of their respective adaptive fit to their environments.

Our study design and experimental setup for the two groups of elephants had several differences resulting from the free‐contact vs. protected‐contact environments in which the savanna and Asian elephants were kept. The first difference in experimental procedures was the fact that the buckets presented to the Asian elephants were nested inside a bucket of the same size that was bolted to the table, while the buckets were held in place on the table by handlers when presented to the savanna elephants. This difference is unlikely to have influenced our results for a few reasons. First, airflow and accessibility to the buckets were not impacted by either method. The bucket bolted to the table in the Asian elephant experiments acted simply as a brace and did not hinder airflow to the top of the bucket in which the odorants were placed. In the case of the savanna elephants where handlers held the buckets in place, handlers never touched the target odors, could not contaminate the buckets, and were rotated randomly to ensure that the elephants could not cue off of them. Moreover, all buckets in both settings were wiped down in between trials to limit differences in external odors that could potentially influence the elephants' choices. While we did not use a separate cloth for each trial, the buckets were wiped down multiple times to reduce any potential identifying odor cues.

An additional difference between the testing setups is that it is possible that the ambient odors of the environments where testing occurred for each species may have differed, which could potentially influence olfactory performance. However, the enclosed box setup used in our experiment minimized exposure to external odors (i.e., elephants sniffed through holes to detect odors trapped inside the box), reducing the likelihood of interference. Additionally, testing was conducted in areas with high airflow, which further limited the concentration of competing ambient odors. Therefore, we believe that background odors did not significantly impact the elephants' ability to detect the target odor.

Another difference was the manner in which the elephants were presented with the buckets. The savanna elephants were instructed to walk up to the table, while the Asian elephants were presented with the buckets on a table that rolled up to them. It is unlikely that this minor difference in procedure would influence the elephants' abilities to identify the target odors because they were both able to touch and interact with the buckets with their trunks for the same amount of time and in the same way. Lastly, the savanna elephants are kept in a more “wild” environment than the Asian elephants. While both species of elephants are barned at night and are used to human contact, the savanna elephants in our study occur in their native range and are allowed to forage naturally during the day, while the Asian elephants are kept in zoo conditions in Syracuse, New York. Additionally, the population of savanna elephants that we used have participated in numerous research projects, a number of which have focused on the elephants' olfactory capabilities. Given the differences in the elephants' captive settings, housing conditions, and prior research experiences, we might anticipate that the Asian elephants, who faced environmental conditions that were most unlike those in which they evolved, would perform worse than savanna elephants in this study. However, our results do not support this prediction. Alternatively, it is possible that the artificial sensory environment in which Asian elephants were housed could have allowed them to perform better than the savanna elephants. Unfortunately, we are unable to determine whether the differences in the environments in which our study populations lived influenced their performance.

Our work is an important step towards comparing the olfactory capabilities of two elephant species that have not shared a common ancestor for at least 4.2 MYA. However, it is possible that some of the variability seen at 50 ppm for Asian elephants and 100 ppm for savanna elephants is a result of our relatively small sample size. This variation in individual abilities highlights the effect of an individual's choices and behaviors in shaping the pattern we see across a system. Olfactory sensitivity and discrimination accuracy can fluctuate within a subject (Phillips and Vallowe 1975), and subjects can have different baseline sensitivities, leading to a single subject's choices impacting the overall results when sample sizes are small. While cognitive studies with elephants typically have a small sample size, we recognize that more research is needed here. In addition to increasing the sample size in future comparisons of olfactory discrimination between elephant species, empirical explorations of the olfactory abilities of African forest elephants are also needed, because they are more closely related to savanna elephants but live in environments more similar to Asian elephants.

Here, even with a limited sample size, we found that both species correctly detected a target odor, but only the Asian elephants maintained their sensitivity in the complex odor environment experiment. The variation in performance between the species observed in our results could be due to differences in the vegetation structure and species diversity, as well as forage quality, in the habitats in which savanna and Asian elephants occur. We recognize access to forage availability and quality may vary across the landscape within the range of each species; thus, forage diversity and olfactory cues could vary between individual elephants. However, our findings highlight the importance of olfaction for both elephant species in the context of foraging. Our study suggests similar olfactory sensitivity in these species, which potentially evolved in a shared ancestor and persisted due to both species' social and foraging needs for olfactory detection. However, our results suggest that they have divergent olfactory discrimination abilities, which are potentially a result of variation in both the woody density and the vegetation species diversity of the habitats in which the two species live. While we only used a single target odor in our experiments (*cis‐3‐Hexenyl acetate*), this VOC is an extremely common green leaf volatile that is emitted by plants globally, including by plants consumed by both species of elephants (Crespo et al. 2013; Koirala et al. 2016; McArthur et al. 2019; Schwarz et al. 2020; Soler et al. 2011). Thus, it is likely that both species of elephants can detect this VOC. However, future studies should explore how the sensitivity and discrimination abilities of savanna and Asian elephants may vary with respect to differing target VOCs and VOC combinations (i.e., natural complex odor bouquets) that may be specific to the environments in which these elephant species live. Moreover, future research should increase sample sizes of tested individuals and focus on the concentration ranges closely above and below our demonstrated sensitivity limits to establish a precise threshold. In addition, future studies should also explore whether olfactory sensitivity and discrimination vary as a function of age and sex to expand our understanding of variation in sensory abilities across different life stages and between individuals. Although our study is fundamentally focused on olfactory sensitivity, our findings may have future applications in conservation and human‐elephant conflict mitigation, such as in the development of scent‐based deterrents to agricultural areas that consider individual‐ and species‐level differences in behavior (Mumby and Plotnik 2018; Plotnik and Jacobson 2022). In conclusion, while we were limited by a small sample size, our results suggest that Asian and savanna elephants may differ in how they detect odors even if their sensitivity to such odors is similar.

## Author Contributions

**Melissa H. Schmitt:** conceptualization (equal), data curation (equal), formal analysis (equal), investigation (equal), methodology (equal), visualization (equal), writing – original draft (equal). **Matthew S. Rudolph:** data curation (equal), formal analysis (equal), investigation (equal), methodology (equal), writing – original draft (equal), writing – review and editing (equal). **Sarah L. Jacobson:** methodology (equal), writing – review and editing (equal). **Joshua M. Plotnik:** conceptualization (equal), formal analysis (equal), investigation (equal), methodology (equal), project administration (equal), supervision (equal), writing – review and editing (equal).

## Ethics Statement

This research was reviewed and approved by both elephant facilities (Adventures with Elephants in South Africa, and the Rosamond Gifford Zoo in New York, USA) and approved by the following ethics committees prior to data collection: Hunter College, City University of New York IACUC (JP‐Categorization Elephants 3/22), the Duke IACUC (#A248‐18‐10) as well as the University of North Dakota IACUC (2504–0020).

## Consent

The authors have nothing to report.

## Conflicts of Interest

The authors declare no conflicts of interest.

## Supporting information


**Table S1:** ece371896‐sup‐0001‐TablesS1‐S4.docx.

## Data Availability

All data can be found on the Zenodo repository (Schmitt et al. 2025).
