# Supplementary material for: Differences in Olfactory Discrimination, but Not Sensitivity, Between African Savanna and Asian Elephants
Source: Ecol Evol. 2025 Aug 11;15(8):e71896. doi: 10.1002/ece3.71896 (PMC12339044; doi:10.1002/ece3.71896)
Supplement: Supplementary file 1 — Table S1: ece371896‐sup‐0001‐TablesS1‐S4.docx. [file ECE3-15-e71896-s001.docx]

**Supporting Information:** Differences in olfactory discrimination, but not sensitivity, between African savanna and Asian elephants

**Authors:** Melissa H. Schmitt, Matthew S. Rudolph, Sarah L. Jacobson, Joshua M. Plotnik

**Table S1:** Summarized savanna elephant data indicating correct choices for Experiment 1. Individual elephants are listed as column headers (e.g., E1 represents Elephant 1), and the treatments—defined by the concentration of the target odor in parts per million (ppm)—are listed as rows.

| **ppm** | **E1** | **E2** | **E3** | **E4** | **E5** |
| --- | --- | --- | --- | --- | --- |
| **5** | 3 | 2 | 1 | 2 | 3 |
| **10** | 3 | 3 | 3 | 5 | 3 |
| **50** | 5 | 4 | 4 | 4 | 4 |
| **100** | 6 | 2 | 3 | 4 | 6 |
| **500** | 4 | 5 | 5 | 5 | 5 |
| **1000** | 3 | 5 | 4 | 3 | 4 |
| **5000** | 5 | ND | 4 | 4 | 6 |
| **10000** | 4 | 4 | 5 | 4 | 6 |
| **50000** | 5 | 6 | 6 | 5 | 6 |
| **100000** | 6 | ND | 6 | 5 | 6 |

**Table S2:** Summarized Asian elephant indicating correct choices data for Experiment 1. Individual elephants are listed as column headers (e.g., E1 represents Elephant 1), and the treatments—defined by the concentration of the target odor in parts per million (ppm)—are listed as rows.

| **ppm** | **E1** | **E2** | **E3** | **E4** | **E5** |
| --- | --- | --- | --- | --- | --- |
| **5** | 2 | 3 | 3 | 2 | 3 |
| **10** | 2 | 3 | 2 | 2 | 3 |
| **50** | 5 | 2 | 5 | 4 | 4 |
| **100** | 4 | 5 | 5 | 6 | 6 |
| **500** | 5 | 4 | 4 | 4 | 4 |
| **1000** | 4 | 4 | 4 | 4 | 4 |
| **5000** | 5 | 3 | 5 | 5 | 5 |
| **10000** | 5 | 3 | 4 | 3 | 5 |
| **50000** | 4 | 5 | 4 | 4 | 4 |
| **100000** | 5 | 4 | 5 | 5 | 4 |

**Table S3:** Summarized savanna elephant data indicating correct choices for Experiment 2. Individual elephants are listed as column headers (e.g., E1 represents Elephant 1), and the treatments—defined by the concentration of the target odor in parts per million (ppm)—are listed as rows.

| **ppm** | **E1** | **E2** | **E3** | **E4** | **E5** |
| --- | --- | --- | --- | --- | --- |
| **5** | 3 | 2 | 3 | 5 | 4 |
| **10** | 3 | 3 | 1 | 4 | 3 |
| **50** | 3 | 3 | 3 | 2 | 4 |
| **100** | 3 | 3 | 4 | 4 | 2 |
| **500** | 3 | 3 | 4 | 3 | 3 |
| **1000** | 3 | 4 | 4 | 6 | 4 |
| **5000** | 5 | 4 | 5 | 5 | 5 |
| **10000** | 5 | ND | 4 | 5 | 5 |
| **50000** | 5 | 6 | 5 | 4 | 5 |
| **100000** | 5 | ND | 5 | 5 | 4 |

**Table S4:** Summarized Asian elephant data indicating correct choices for Experiment 2. Individual elephants are listed as column headers (e.g., E1 represents Elephant 1), and the treatments—defined by the concentration of the target odor in parts per million (ppm)—are listed as rows.

| **ppm** | **E1** | **E2** | **E3** | **E4** | **E5** |
| --- | --- | --- | --- | --- | --- |
| **5** | 1 | 2 | 3 | 3 | 3 |
| **10** | 2 | 2 | 3 | 3 | 2 |
| **50** | 5 | 2 | 4 | 6 | 4 |
| **100** | 5 | 6 | 6 | 5 | 4 |
| **500** | 5 | 4 | 5 | 4 | 6 |
| **1000** | 4 | 6 | 6 | 5 | 5 |
| **5000** | 4 | 4 | 5 | 4 | 5 |
| **10000** | 4 | 4 | 4 | 3 | 5 |
| **50000** | 5 | 5 | 6 | 5 | 5 |
| **100000** | 4 | 5 | 4 | 6 | 5 |
